# Height and cancer incidence in the Million Women Study: prospective cohort, and meta-analysis of prospective studies of height and total cancer risk

**DOI:** 10.1016/S1470-2045(11)70154-1

**Published:** 2011-08

**Authors:** Jane Green, Benjamin J Cairns, Delphine Casabonne, F Lucy Wright, Gillian Reeves, Valerie Beral, for the Million Women Study collaborators

**Affiliations:** a Cancer Epidemiology Unit, University of Oxford, UK; b Unit of Infections and Cancer (UNIC), Institut Català d'Oncologia, Barcelona, Spain

## Abstract

**Background:**

Epidemiological studies have shown that taller people are at increased risk of cancer, but it is unclear if height-associated risks vary by cancer site, or by other factors such as smoking and socioeconomic status. Our aim was to investigate these associations in a large UK prospective cohort with sufficient information on incident cancer to allow direct comparison of height-associated risk across cancer sites and in relation to major potential confounding and modifying factors.

**Methods:**

Information on height and other factors relevant for cancer was obtained in 1996–2001 for middle-aged women without previous cancer who were followed up for cancer incidence. We used Cox regression models to calculate adjusted relative risks (RRs) per 10 cm increase in measured height for total incident cancer and for 17 specific cancer sites, taking attained age as the underlying time variable. We also did a meta-analysis of published results from prospective studies of total cancer risk in relation to height.

**Findings:**

1 297 124 women included in our analysis were followed up for a total of 11·7 million person-years (median 9·4 years per woman, IQR 8·4–10·2), during which time 97 376 incident cancers occurred. The RR for total cancer was of 1·16 (95% CI 1·14–1·17; p<0·0001) for every 10 cm increase in height. Risk increased for 15 of the 17 cancer sites we assessed, and was statistically significant for ten sites: colon (RR per 10 cm increase in height 1·25, 95% CI 1·19–1·30), rectum (1·14, 1·07–1·22), malignant melanoma (1·32, 1·24–1·40), breast (1·17, 1·15–1·19), endometrium (1·19, 1·13–1·24), ovary (1·17, 1·11–1·23), kidney (1·29, 1·19–1·41), CNS (1·20, 1·12–1·29), non-Hodgkin lymphoma (1·21, 1·14–1·29), and leukaemia (1·26, 1·15–1·38). The increase in total cancer RR per 10 cm increase in height did not vary significantly by socioeconomic status or by ten other personal characteristics we assessed, but was significantly lower in current than in never smokers (p<0·0001). In current smokers, smoking-related cancers were not as strongly related to height as were other cancers (RR per 10 cm increase in height 1·05, 95% CI 1·01–1·09, and 1·17, 1·13–1·22, respectively; p=0·0004). In a meta-analysis of our study and ten other prospective studies, height-associated RRs for total cancer showed little variation across Europe, North America, Australasia, and Asia.

**Interpretation:**

Cancer incidence increases with increasing adult height for most cancer sites. The relation between height and total cancer RR is similar in different populations.

**Funding:**

Cancer Research UK and the UK Medical Research Council.

## Introduction

Tall people are at increased risk of cancer. Increasing cancer risk with increasing adult height has been reported for all cancers combined and for several common cancers, such as those of the breast, ovary, prostate, and large bowel.^1^, ^2^, ^3^, ^4^, ^5^, ^6^, ^7^ Evidence is limited, however, for incident, rather than fatal, disease and for less common cancer sites. Moreover, it is not clear to what extent height-associated risks vary by cancer site, or how other factors, such as smoking and socioeconomic status, affect these associations.^8^, ^9^ Because the range of height in a given population is usually narrow, large numbers of events are needed for reliable estimation of risk. Therefore we report here on the relation between height and cancer incidence in a prospective cohort study of more than 1 million middle-aged women in the UK. We also did a meta-analysis of published results from prospective studies on the relation between height and total cancer incidence or mortality.

## Methods

### Participants

Between 1996 and 2001, 1·3 million middle-aged women invited to attend the UK's National Health Service (NHS) Breast Screening Programme completed a Million Women Study recruitment questionnaire, which asked, among other things, about social, demographic, and lifestyle factors, including current height and weight. Of women who answered a study questionnaire in 2006–07, a sample selected at random (on the basis of day of birth) were asked in 2006–09 to have their height measured by their family doctor: 3762 women did so. In this validation sample, the correlation between measured and reported heights was excellent (Pearson correlation coefficient 0·88).

For **study protocols and questionnaires** see http://www.millionwomenstudy.org/

All participants gave written consent to take part in our study, and approval was obtained from the Oxford and Anglia Multi-Centre Research Ethics Committee. All study participants have a unique NHS number and are automatically followed up for death, emigration, and cancer registration through the NHS central registers with that number and other identifying details. The registers regularly provide study investigators with information on the date of any such event in participants, and code the underlying cause of death and cancer site with the International Classification of Diseases, 10th revision (ICD-10).^10^ Follow-up is complete for over 99% of study participants.

### Procedures

Our main endpoints were incident invasive cancer at 17 individual sites with at least 1000 incident cases: mouth and pharynx (ICD-10 C00-C14), oesophagus (C15), stomach (C16), colon (C18), rectum (C19-20), pancreas (C25), lung (C34), malignant melanoma (C43), breast (C50), endometrium (C54), ovary (C56), kidney (C64), bladder (C67), central nervous system (C70–72, D32, 33, 42, and 43), non-Hodgkin lymphoma (C82-85), multiple myeloma (C90), and leukaemia (C91-95). We included all other invasive cancers (the remaining ICD-10 C codes, except non-melanoma skin cancer [C44]) as “other and unspecified” cancers.

We defined smoking-related cancers as those for which the International Agency for Research on Cancer (IARC) has concluded there is sufficient evidence of carcinogenicity in human beings in relation to active tobacco smoking:^11^, ^12^ of the sites listed above, mouth and pharynx, oesophagus, stomach, colorectum, pancreas, lung, mucinous tumours of the ovary, kidney, and myeloid leukaemia (C92), and, additionally, liver (C22), larynx, nasal cavity and nasal sinuses (C30-32), cervix (C53), and urinary tract, including renal pelvis and ureter (C65, 66, 68). When comparing smoking-related and other cancers, we excluded from our analysis cancers of ill-defined and unspecified sites, which might include some smoking-related cancers (ICD-10 C26, C39, C57, C76-80 and C95-96), and cancers of the ovary (for a substantial proportion of which histological subtype was not known, and which might have included mucinous tumours).

Height was reported by participants at recruitment in feet and inches, and converted to centimetres for our analysis. For the analyses, women were divided into six categories of reported height (<155 cm [reference group], 155–159·9 cm, 160–164·9 cm, 165–169·9 cm, 170–174·9 cm, and 175 cm and taller); we took the average height in each of these categories to be the mean measured height in that category in the sample whose height was measured in 2006–09. Where appropriate, mean measured heights are reported standardised to the distribution of self-reported heights within the whole population, or relevant subgroup.

We excluded women from our analyses if they had any type of cancer other than non-melanoma skin cancer (ICD10 C44) registered before recruitment and if they did not have valid information on height at recruitment (including a small proportion, about 0·05% whose reported height was <120 cm or >200 cm). For analyses including endometrial and/or cervical cancers, we excluded women if they reported a hysterectomy at recruitment, or if their hysterectomy status was unknown; similarly, for analyses of ovarian cancer, we excluded women if they reported a bilateral oophorectomy at recruitment, or if their oophorectomy status was unknown.

We calculated woman-years from the date of recruitment to the date of first cancer registration (at any site), death, or the last date of follow-up, whichever was first. For analyses of cancer incidence, the last date of follow-up was Dec 31, 2008, for the UK regions of East Anglia and South West; June 30, 2008, for Oxford, Thames, West Midlands, and North West (Mersey); and Dec 31, 2007, for Northern and Yorkshire, Trent, North West (Manchester and Lancashire), and Scotland.

### Statistical analysis

We used Cox regression models to estimate relative risks (RRs) and CIs in relation to height at recruitment, taking attained age as the underlying time variable. We stratified all analyses by age at recruitment (<52, 53–55, 56–58, 59–61, 62–64, ≥65 years) and region (ten regions covered by ten cancer registries), and adjusted, as appropriate, for quintiles of socioeconomic group (based on Townsend deprivation score^13^), body-mass index (<22·5, 22·5–24·9, 25·0–27·4, 27·5–29·9, ≥30 kg/m^2^), strenuous exercise (less than once a week, once a week or more), alcohol consumption (none, ≤2 units per week, ≥3 units per week), smoking (never, past, current 1–14 cigarettes per day, current ≥15 cigarettes per day), age at menarche (<13, 13, ≥14 years), parity (0, 1–2, ≥3 full-term pregnancies), and age at first birth (<25, ≥25 years). We assigned missing values of adjustment variables a separate category and did sensitivity analyses restricted to women with available information on all adjustment variables. Information on all variables was that provided at recruitment.

We calculated the RR per 10 cm increase in height as a trend across the six category means using the measured mean height in each category of reported height.^14^ We assessed heterogeneity of trends in RRs between different cancer sites with a (χ^2^) contrast test, under the survival analysis assumptions that estimates at each cancer site are asymptotically normally distributed and, because of censoring at first cancer diagnosis at any site, uncorrelated (and that therefore site-specific estimates account for competing risks of cancers at other sites).^15^

Where two categories of exposure are compared (as in the text) conventional CIs are given. For analyses of total cancer, where more than two categories are compared (as in the figures), floated CIs (FCIs) were estimated by treating the RRs as floating absolute risks (FARs).^16^, ^17^ Use of floated methods allows valid comparisons to be made between any two exposure groups, even if neither is the baseline group. All results are presented in the text with 95% CIs, but for analyses by cancer site, when multiple RRs were estimated, 99% CIs are given in the figures.

Where we present results in the form of plots, the RRs and their corresponding FCIs or CIs are represented by squares and lines with the area of each square inversely proportional to the variance of the logarithm of the corresponding RR. This shows the amount of statistical information involved.

### Meta-analysis

We identified published prospective studies of adult height and risk of total cancer (incidence or mortality) through electronic searches of published work (Medline and Embase, up to April, 2011) with combinations of the search terms “height”, “body size”, “anthropometr*”, “neoplasms”, “mortality”, and “risk factors”, and through cited references in identified papers. We did not limit our searches by study size, source of study population, date, or language of publication. We included in our meta-analysis all identified studies with published age-adjusted RR and 95% CIs for total cancer per 10 cm increase in height, or with sufficient published data to allow estimation of such RRs. Data were extracted independently by two researchers (JG and BJC). Where only categorical results were published, we calculated the trend in RR per 10 cm increase in height using the mean height in each height category (estimated, where necessary, as category midpoints, or by other methods),^18^ assuming linearity of log RRs and with a summary trend estimate obtained by the method of generalised least squares.^19^ We combined results for subgroups of total cancer (eg, smoking-related and other cancers) by inverse-variance weighted least squares, where necessary. For each study we used the most fully adjusted RR available. Study-specific RRs were combined to give summary RRs with weights proportional to the inverse of the variance. Where the mean year of birth of the study population was not given, we made an estimate with the average age at, and year of, study recruitment. Where necessary we estimated the mean height of the study population using mean height within categories and the categorical distribution of heights within the study population. For all analyses we used Stata (version 11.1).

### Role of the funding source

The funding sources did not influence the design of the study, the collection, interpretation and analysis of the data, the preparation of this report, or the decision to publish. BJC, DC, and JG had access to the raw data for the study. The corresponding author had full access to all data and the final responsibility for the decision to submit for publication.

## Results

The 1 297 124 women included in our analysis had a mean age at recruitment of 56·1 years (SD 4·9) and an average year of birth of 1942. The median length of follow-up was 9·4 years per woman (IQR 8·4–10·2 years), for a total of 11·7 million person-years, during which 97 376 incident cancers were notified.

Table 1 shows characteristics of the study population, including measured height, by six categories of height reported at recruitment. Taller women tended to be of higher socioeconomic status, to drink more alcohol, to be more active, to have a later age at menarche, to have fewer children, and to have their first child later in life than shorter women. Taller women were less likely to be obese or to be current smokers. Based on heights measured in the validation sample, the mean height in the study population was 160·9 cm (SD 6·4).

**Table 1:** Baseline characteristics by height and follow-up for incident cancer in the Million Women Study

		Height in cm*						**All women**
		<155	155	160	165	170	≥175	
Mean measured height (SD)		152·8 (4·1)	156·5 (2·3)	160·4 (2·9)	164·9 (2·9)	169·0 (2·9)	173·8 (4·3)	160·9 (6·4)†
Characteristics at recruitment								
	Number of women	233 516	196 773	388 515	288 893	143 289	46 138	1 297 124
	Mean age, years (SD)	56·3 (4·9)	56·2 (4·9)	56·2 (4·9)	56·0 (4·8)	56·0 (4·8)	55·8 (4·8)	56·1 (4·9)
	Socioeconomic status, n (%) in lowest quintile	59 220 (26%)	42 862 (22%)	73 119 (19%)	48 190 (17%)	23 262 (16%)	7 664 (17%)	19·7
	Current smokers, n (%)	50 775 (23%)	40 500 (22%)	72 763 (20%)	51 678 (19%)	26 147 (19%)	8 369 (19%)	20·5
	Alcohol intake, n (%) ≥7 units per week	47 138 (20%)	43 324 (22%)	92 126 (24%)	73 597 (26%)	36 742 (26%)	11 734 (26%)	23·7
	Body-mass index, n (%) BMI ≥30	54 550 (25%)	38 493 (20%)	65 622 (18%)	42 004 (15%)	18 370 (13%)	5 320 (12%)	18·0
	Strenuous exercise, n (%) once a week or more	76 917 (35%)	69 607 (37%)	147 103 (39%)	116 614 (42%)	58 339 (42%)	18 699 (42%)	39·0
	Age at menarche, n (%) ≥14 years	79 858 (35%)	69 718 (36%)	139 607 (37%)	108 550 (38%)	57 852 (41%)	20 176 (45%)	37·4
	Parity, n (%) nulliparous	22 827 (10%)	19 149 (10%)	40 296 (10%)	33 267 (12%)	17 985 (13%)	6 900 (15%)	10·8
	Number of full-term pregnancies, n (%) with three or more	82 436 (35%)	67 118 (34%)	127 826 (33%)	91 287 (32%)	44 074 (31%)	13 335 (29%)	32·9
	Age at first birth, n (%) ≥25 years	67 250 (33%)	61 042 (35%)	129 031 (38%)	103 017 (41%)	52 677 (43%)	17 492 (46%)	38·2
	Postmenopausal, n (%)	162 551 (81%)	136 544 (81%)	269 384 (81%)	197 618 (80%)	97 855 (80%)	30 900 (79%)	80·5
	Ever use of oral contraceptives, n (%)	133 979 (58%)	114 105 (59%)	228 669 (60%)	173 520 (61%)	85 522 (60%)	27 571 (60%)	59·5
	Current use of HRT, n (%)	75 151 (33%)	63 865 (33%)	128 891 (34%)	98 086 (34%)	48 516 (34%)	15 637 (34%)	33·6
Follow-up for cancer incidence								
	Woman-years, millions	2·1	1·8	3·5	2·6	1·3	0·4	11·7
	Number of incident cancers	15 792	14 213	28 806	22 571	11 902	4 092	97 376

* The categories of height are those reported at recruitment, and mean values are those measured in a randomly selected sample.

† Standardised to the distribution of categories of self-reported height in our whole analysis population.

**Figure 1:**
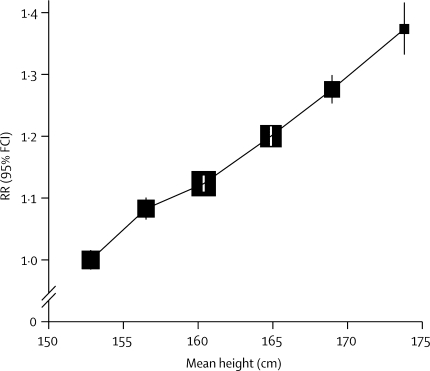
Relative risks (RRs) and 95% floated CIs (FCIs) for total incident cancer, by height RRs are adjusted for age, region, socioeconomic status, smoking, alcohol intake, body-mass index, strenuous exercise, age at menarche, parity, and age at first birth, and are plotted against the mean measured height in each category.

Total cancer incidence rose with increasing height (table 2). Comparing women in the tallest group with those in the shortest group (a difference of 21 cm: mean measured heights 174 cm and 153 cm), the adjusted RR for total incident cancer was 1·37 (95% CI 1·33–1·42; p<0·0001). The RR for total cancer was 1·16 (1·14–1·17; p<0·0001) per 10 cm increase in height (figure 1).

**Table 2:** Relative risks (RRs) and 95% floated CIs (FCIs) for total cancer incidence, by category of height reported at recruitment (mean measured height)

	**Women**	**Incident cancers**	**RR (95% FCI)**
<155 cm (mean 152·8 cm)	233 516	15 792	1·00 (0·98–1·02)
155 cm (mean 156·5 cm)	196 773	14 213	1·08 (1·07–1·10)
160 cm (mean 160·4 cm)	388 515	28 806	1·12 (1·11–1·14)
165 cm (mean 164·9 cm)	288 893	22 571	1·20 (1·18–1·22)
170 cm (mean 169·0 cm)	143 289	11 902	1·28 (1·25–1·30)
≥175 cm (mean 173·8 cm)	46 138	4092	1·37 (1·33–1·42)

Analysis stratified by age at recruitment and region and adjusted for socioeconomic status, smoking, alcohol intake, body mass index, strenuous exercise, age at menarche, parity, and age at first birth.

**Figure 2:**
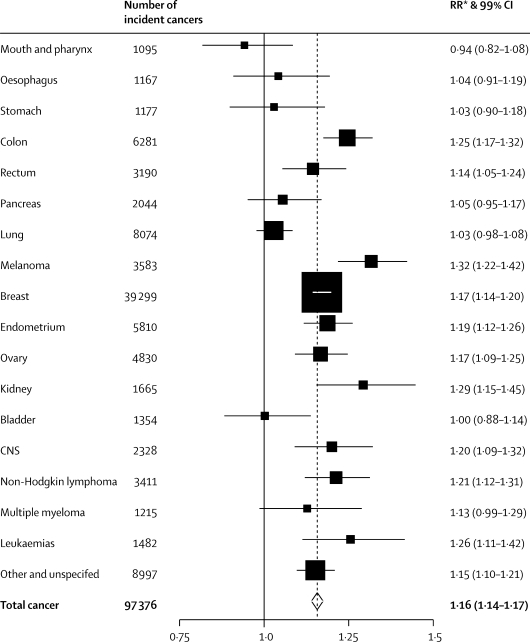
Relative risks (RRs) and 99% CIs per 10 cm increase in height for incident cancer at 17 specific sites and for total cancer The doted line represents the RR per 10 cm increase in height for total cancer. *RRs are adjusted for age, region, socioeconomic status, smoking, alcohol intake, body-mass index, strenuous exercise, age at menarche, parity, and age at first birth.

Figure 2 shows the RRs per 10 cm increase in height for the 17 separate cancer sites we assessed, for all other cancers and for total cancer. The height-associated RRs are greater than 1·0 for 15 of the 17 specific sites, and are significantly increased for ten specific sites and for the group of other and unspecified cancers: colon (RR per 10 cm increase in height 1·25, 95% CI 1·19–1·30), rectum (1·14, 1·07–1·22), malignant melanoma (1·32, 1·24–1·40), breast (1·17, 1·15–1·19), endometrium (1·19, 1·13–1·24), ovary (1·17, 1·11–1·23), kidney (1·29, 1·19–1·41), central nervous system (1·20, 1·12–1·29), non-Hodgkin lymphoma (1·21, 1·14–1·29), leukaemia (1·26, 1·15–1·38), and other cancers (1·15, 1·11–1·20). For no cancer site was there a significant decrease in risk with increasing height. There is heterogeneity across cancer sites (contrast test χ^2^ [17 degrees of freedom]=115·2; p<0·0001) mostly because of the greater than average increase in risk with increasing height for colon cancer and for malignant melanoma, and the lower than average risk for lung cancer. Breast cancer accounts for half of incident cancers in our study and the results for breast cancer therefore dominate the overall results. However, the overall RR of incident cancer in relation to height was not materially altered when we excluded breast cancer cases from our analysis (RR per 10 cm increase in height 1·15, 95% CI 1·13–1·16).

We adjusted our results in Figure 1, Figure 2 and in table 2 by age, region, socioeconomic status, smoking, alcohol, body-mass index, physical activity, age at menarche, parity, and age at first birth. Table 3 shows the effect of adjustment by potential confounding variables on the RR for total cancer per 10 cm increase in height in an analysis restricted to the 1 087 489 women with full information on all adjustment variables. Compared with the risk with adjustment for age and region only (RR 1·14, 95% CI 1·13–1·15), additional adjustment by the remaining factors increases the RR slightly to 1·16 (1·15–1·18).

**Table 3:** Relative risks (RRs) and 95% CIs per 10 cm increase in height, for total incident cancer: effect of adjustment by various factors

		**RR (95% CI)**
Adjusted by age and region only		1·14 (1·13–1·15)
Additionally adjusted separately by		
	Socioeconomic status	1·15 (1·13–1·16)
	Alcohol	1·14 (1·13–1·15)
	Smoking	1·15 (1·13–1·16)
	Body-mass index	1·15 (1·14–1·17)
	Strenuous exercise	1·14 (1·13–1·16)
	Age at menarche	1·14 (1·13–1·16)
	Parity	1·13 (1·12–1·15)
	Age at first birth	1·14 (1·13–1·15)
Adjusted simultaneously for all of the above		1·16 (1·15–1·18)

Analysis restricted to 1 087 489 women (81 797 with cancer) with information on all adjustment variables.

**Figure 3:**
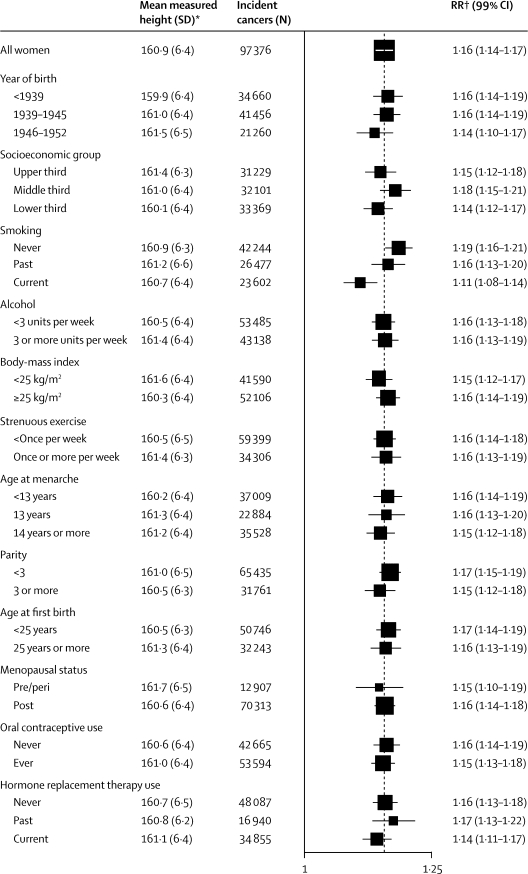
Relative risks (RRs) and 99% CIs per 10 cm increase in height for all incident cancer, by various characteristics at recruitment The dotted line represents the RR per 10 cm increase in height for all women. *Standardised to the distribution of self-reported heights within each subgroup of the whole study population. †RRs are adjusted as appropriate for age, region, socioeconomic status, smoking, alcohol intake, body-mass index, strenuous exercise, age at menarche, parity, and age at first birth.

**Figure 4:**
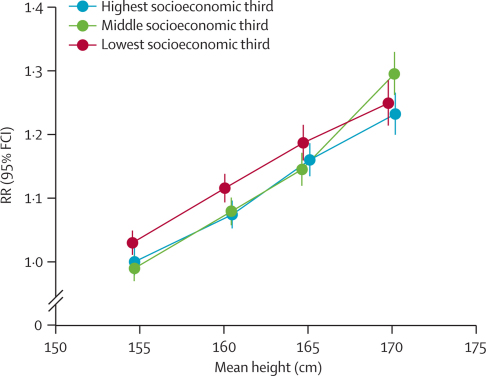
Relative risks (RRs) and 95% floated CIs (FCIs) for all incident cancer in relation to height, and by socioeconomic status The baseline category (RR=1·0) is women shorter than 160 cm from the highest socioeconomic group. RRs are adjusted for age, region, smoking, alcohol intake, body-mass index, strenuous exercise, age at menarche, parity, and age at first birth. RRs are plotted against the mean measured height in each category of height (<160 cm, 160–165 cm, 165–170 cm, ≥170 cm), within categories of socioeconomic status.

Figure 3 shows the RR for total cancer per 10 cm increase in height, and the mean measured height, in subgroups of women defined by their year of birth, socioeconomic status, smoking status, alcohol consumption, body-mass index, physical activity, age at menarche, parity, age at first birth, menopausal status, and use of oral contraceptives and hormone replacement therapy. As we expected, women born before 1939 were shorter than women born in 1946 or later (mean measured height 159·9 *vs* 161·5 cm), as were women from the lowest compared to the highest socioeconomic tertile (160·1 *vs* 161·4 cm). However, the height-associated RR for total cancer did not vary significantly by these or by most other characteristics. Figure 4 shows this lack of variation by socioeconomic status. Although the risk for total cancer is somewhat higher in women in the lowest tertile of socioeconomic status, the pattern of risk by height is similar in all three tertiles. Of the 12 personal characteristics we assessed, only smoking status substantially modified the size of the height-related RRs (figure 3). The RR per 10 cm greater height was 1·19 (95% CI 1·17–1·21) in never smokers, but only 1·11 (1·08–1·14) in current smokers (p<0·0001 for heterogeneity).

**Figure 5:**
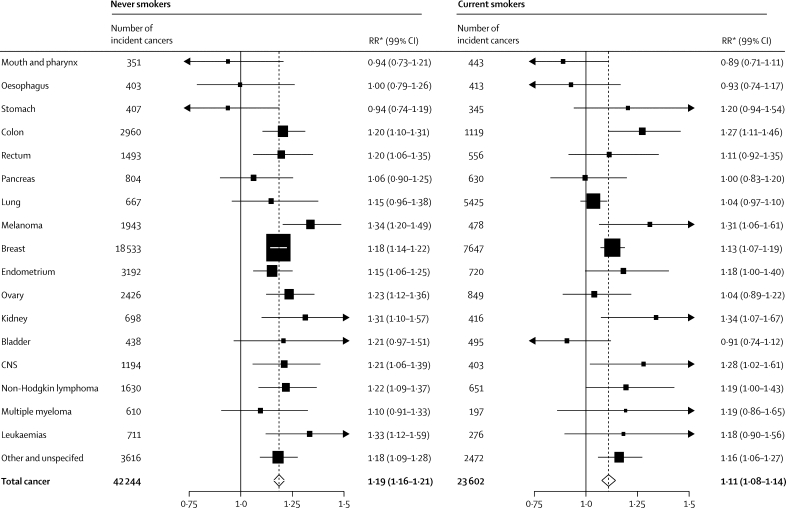
Relative risks (RRs) and 99% CIs per 10 cm increase in height, for all incident cancer and for incident cancer at 17 specific sites, in never and current smokers Dotted lines represent the RR for total cancer. *RRs are adjusted for age, region, socioeconomic status, alcohol intake, body-mass index, strenuous exercise, age at menarche, parity, and age at first birth.

Figure 5 shows the RRs per 10 cm increase in height by cancer site in never smokers and in current smokers (results in past smokers are uninterpretable, because they are a heterogeneous group with a wide range of times since last smoking). The mix of cancers differs in the two groups with, as expected, a higher proportion of women with lung and other smoking-related cancers in current smokers than in never smokers. In never-smokers, heterogeneity across cancer sites was substantially weaker (p=0·004) than in current smokers (p<0·0001).

**Figure 6:**
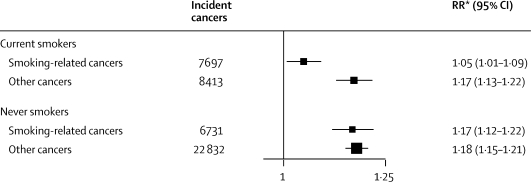
Relative risks (RRs) and 95% CIs per 10 cm increase in height for incident smoking-related and other specified cancers, in never and in current smokers *RRs are adjusted for age, region, socioeconomic status, alcohol intake, body-mass index, strenuous exercise, age at menarche, parity, and age at first birth.

For smoking-related cancers, the RR per 10 cm greater height was substantially smaller in current smokers than in never smokers (1·05 *vs* 1·17, p for difference=0·0004; figure 6). By contrast, for other specified cancers height-associated RRs were similar in current smokers and in never smokers, and close to our estimate for smoking-related cancers in never smokers (figure 6).

Published evidence suggests that current smoking is not a strong risk factor for colorectal cancer^20^ and the number of these cancers is large, so we undertook a sensitivity analysis with colorectal cancer classed as not related to smoking (ie, as in the latest full report on smoking and cancer available from IARC^11^). The overall pattern of RRs remained similar, with lower risk for smoking-related cancers than for other cancers in current smokers, although the difference between these risks was reduced (RR per 10 cm height 1·02, 95% CI 0·97–1·06, in current smokers and 1·10, 1·03–1·17, in never smokers; p for difference=0·05); for other specified cancers, risks remained similar to those in our main analysis (RRs 1·18, 1·14–1·22, in current smokers and 1·19, 1·16–1·21, in never smokers).

Because breast cancer dominates our findings, we repeated our analyses shown in figure 3 separately for the five most common cancers in our study: breast, lung, colon, endometrium, and ovary, and for the remaining cancers. Overall, we did not identify significant heterogeneity, by the 12 factors we show in figure 3, for these cancer sites (χ^2^ test for heterogeneity aggregated across all characteristics: colon p=0·7, lung p=0·2, breast p=0·3, endometrium p=0·5, ovary p=0·2, remaining cancers p=0·2).

**Figure 7:**
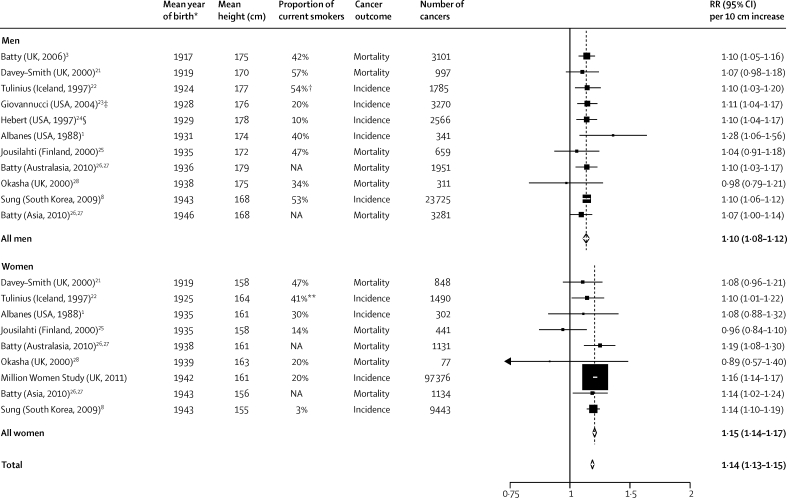
Meta-analysis of results from prospective studies: study-specific and summary relative risks (RRs) and 95% CIs for all cancer per 10 cm increase in height The dotted lines represent the summary RRs. NA=not available. *Mean years of birth estimated as necessary. †Includes 24% (men) and 2% (women) pipe or cigar smokers. ‡Category midpoints used to estimate mean heights in height categories. §Method of Chêne and Thompson^18^ used to estimate mean heights in height categories.

Because there was no strong variation by cancer site in our study except in smokers, we did a meta-analysis of published studies of all-cancer risk, noting for each study the proportion of current smokers in the study population. Figure 7 shows details of our study together with ten other prospective studies^1^, ^3^, ^8^, ^21^, ^22^, ^23^, ^24^, ^25^, ^26^, ^27^, ^28^ that have published results in such a way as to allow estimation of the RR of total cancer incidence or mortality per 10 cm increase in height. The populations covered include men and women from Asia, Australasia, Europe, and North America, with mean years of birth ranging over three decades (1917 to 1946), and with mean heights ranging over 24 cm (155 to 179 cm). The overall increase in RR per 10 cm greater height is 1·14 (95% CI 1·13–1·15). There was no significant heterogeneity between the results from studies in men (*I*^2^ for heterogeneity 0%, p=0·9) or between those in women (*I*^2^ for heterogeneity 31%, p=0·2), but there was a slightly lower height-associated RR in men than in women (1·10 *vs* 1·15, p for difference <0·0001). When we excluded the findings of our study, the summary RR in women was slightly reduced (summary RR per 10 cm greater height 1·13, 95% CI 1·10–1·16; *I*^2^ for heterogeneity 25%; p=0·2), and there was no longer significant heterogeneity between studies in men and those in women (p for difference=0·1). In our meta-analysis we included studies of cancer mortality as well as those of cancer incidence. All of the mortality studies we included provided RRs adjusted for at least one measure of socioeconomic status, which should have minimised potential confounding due to the relation in many populations between socioeconomic status and cancer survival.^29^

## Discussion

We identified a clear and highly significant trend of increasing cancer risk with increasing height in this large prospective study of UK women, with RR for total incident cancer of 1·16 (99% CI 1·14–1·17) for every 10 cm greater height. The magnitude of the height-associated increase in cancer risk was similar for women with different years of birth, from different socioeconomic groups, and across subgroups defined by alcohol intake, body-mass index, physical activity, age at menarche, parity, age at first birth, menopausal status, and use of oral contraceptives or hormone replacement therapy. By contrast, current smokers had a lower RR for total cancer incidence per 10 cm increase in height than never smokers, and this was largely because the height-associated cancer RRs in smokers were lower for smoking-related than for other cancers. In never smokers, there was only weak variation in height-related risk across 17 cancer sites.

This study included more than 97 000 incident cancers. Each of the 17 most common specific sites we assessed included 1000 or more cancers, and together they constitute some 90% of total incident cancers in our study population. Most previous studies had limited statistical power to study site-specific cancer risk and tended to focus on a few common cancer sites, and our results for the common cancers are consistent with their findings.^2^, ^4^, ^5^, ^6^, ^7^

All study participants were routinely linked to records of the NHS central registers and details of every incident cancer and death were coded before notification to the study investigators, thus providing complete and non-differential ascertainment of cancer incidence during follow-up. Women were categorised by the height reported at recruitment, and height was measured some years later in a sample of just over 3700 women. There was excellent correlation between self-reported and measured height, consistent with previous findings for height and some other anthropometric variables in this cohort.^30^, ^31^ Nevertheless, we corrected for measurement error, and for changes in height over time, by use of the mean measured height in each category to calculate RR per 10 cm increase in height.

As expected, the average height of women in this population was slightly greater the more recently they were born and with increasing socioeconomic status (figure 3). To minimise potential confounding by these and other factors relevant for cancer, all analyses compared women of a similar age, region of residence, socioeconomic group, age at menarche, parity, age at first birth, body-mass index, physical activity, smoking status, and alcohol consumption. Because of the large size of this study we were also able to undertake subgroup analyses by 12 potential confounding factors, in particular by socioeconomic status. Women in higher socioeconomic groups are on average taller (table 1), and socioeconomic status is related to total cancer incidence (figure 4),^29^ yet the association between height and risk of cancer was similar for women of low, medium, and high socioeconomic status. As in other studies that could adjust for a range of potential confounding factors, our results suggest that the relation between height and cancer risk is not due to other known risk factors for cancer.^9^

Our findings show that the height-related RR of cancer was lower for smoking-related cancers than for other cancers, but only in current smokers. In accordance with our findings Kabat and colleagues^32^ have reported that lung cancer incidence in the Women's Health Initiative study showed a stronger association with height in never smokers than in current or past smokers. Our test for potential modification of height-related cancer risks by smoking status used a multiplicative model. However, on an absolute scale there is little difference between current and never smokers in the excess cancer incidence rates. Smoking-related cancers are more common in current smokers than in never smokers, with age-standardised incidence rates of 599 and 176, respectively per 100 000 women per year, in this cohort. The estimated excess age-standardised incidence rate for every 10 cm increase in height for smoking-related cancers is about 30 per 100 000 women per year, both in current and in never smokers (599 × 0·05 for current smokers and 176 × 0·17 for never smokers).

We found no other modification of height-associated RR by the 11 other factors we assessed, either for total cancer, or separately for the five most common cancers (breast, lung, colon, endometrium, and ovary). However, even in our large study we had limited power to assess modification of height-related risk by these factors.

There was little variation in height-associated RRs at specific cancer sites in never smokers, in whom the effect of height on cancer risk is free from modification by smoking. Most other studies have not made direct comparisons across cancer sites or between smokers and non-smokers. In general, studies have found taller people to be at increased risk of a range of cancers with varying causes, with no individual cancer site consistently identified as showing no association.^2^, ^4^, ^5^, ^6^, ^7^ Our finding of differences in height-related RR between smokers and never smokers might provide an explanation for some reported inconsistencies in height-associated risk for smoking-related cancers.^2^

Our meta-analysis of height and total cancer risk shows that findings are very consistent for incidence and for mortality, and in populations from Europe, North America, Asia, and Australasia with mean years of birth ranging over 30 years, and with mean heights ranging from 155 cm to 179 cm. Women in these studies were less likely than men to be current smokers (figure 7) and this might partly explain the slightly higher height-associated RR in women than in men in our meta-analysis. The overall result in women is also strongly weighted by the results from the Million Women Study, in which there has been allowance for measurement error, and more extensive adjustment than in the other studies, both of which tended to increase the estimated RR. As in any meta-analysis of published data, our findings need to be interpreted in the knowledge that other studies with relevant data might not have published their results.

The similarity of the height-associated RR for different cancers and in different populations suggests that a basic common mechanism, possibly acting in early life, might be involved.^8^ Adult height reaches its maximum between the ages of 20 and 30 years. Variation in height relates to genetic and environmental influences acting mostly in the first 20 years, or so, of life; environmental factors, including childhood nutrition and infections, are believed to predominate.^33^, ^34^, ^35^, ^36^ Hormone levels, especially of growth factors such as insulin-like growth factors (IGFs), both in childhood and in adult life, might be relevant.^2^, ^9^ Circulating levels of IGFs in adulthood and childhood affect cancer risk;^37^, ^38^, ^39^, ^40^ IGF-I levels in childhood and adolescence are strongly related to skeletal growth,^38^ and levels in adulthood, although less strongly, to adult height.^41^, ^42^ Another possibility is that height predicts cancer risk because taller people have more cells (including stem cells), and thus a greater opportunity for mutations leading to malignant transformation.^43^, ^44^ Height might thus be related to cancer risk through increased cell turnover mediated by growth factors, or through increased cell numbers.

The relation between height and cancer risk might underlie part of the difference in cancer incidence between populations, and changes in cancer incidence over time. Adult height in European populations has increased by about 1 cm per decade throughout the 20th century.^33^, ^45^, ^46^ The increase in adult height during the past century could thus have resulted in an increase in cancer incidence some 10–15% above that expected if population height had remained constant. This assumes, of course, that the effect of height is independent of changes in other risk factors.

## Web Extra Material

Supplementary webappendix
