# Supplementary material for: Height and cancer incidence in the Million Women Study: prospective cohort, and meta-analysis of prospective studies of height and total cancer risk
Source: Lancet Oncol. 2011 Aug;12(8):785–94. doi: 10.1016/S1470-2045(11)70154-1 (PMC3148429; doi:10.1016/S1470-2045(11)70154-1)
Supplement: Supplementary webappendix [file mmc1.pdf]

THE LANCET Oncology

Supplementary webappendix

This webappendix formed part of the original submission and has been peer reviewed. We post it as supplied by the authors.

Supplement to: Green J, Cairns BJ, Casabonne D, et al. Height and cancer incidence in the Million Women Study: prospective cohort, and meta-analysis of prospective studies of height and total cancer risk. *Lancet Oncol* 2011; published online July 21. DOI:10.1016/S1470-2045(11)70154-1.

Height and cancer incidence in the Million Women Study, and meta-analysis of prospective studies of height and total cancer risk

Appendix: Million Women Study Collaborators

Steering Committee
Emily Banks, Valerie Beral, Ruth English, Jane Green, Julietta Patnick, Richard Peto, Gillian Reeves, Martin Vessey, and Matthew Wallis.

NHS Breast Screening Centres collaborating in the Million Women Study (in alphabetical order):
Avon, Aylesbury, Barnsley, Basingstoke, Bedfordshire & Hertfordshire, Cambridge & Huntingdon, Chelmsford & Colchester, Chester, Cornwall, Crewe, Cumbria, Doncaster, Dorset, East Berkshire, East Cheshire, East Devon, East of Scotland, East Suffolk, East Sussex, Gateshead, Gloucestershire, Great Yarmouth, Hereford & Worcester, Kent (Canterbury, Rochester, Maidstone), Kings Lynn, Leicestershire, Liverpool, Manchester, Milton Keynes, Newcastle, North Birmingham, North East Scotland, North Lancashire, North Middlesex, North Nottingham, North of Scotland, North Tees, North Yorkshire, Nottingham, Oxford, Portsmouth, Rotherham, Sheffield, Shropshire, Somerset, South Birmingham, South East Scotland, South East Staffordshire, South Derbyshire, South Essex, South Lancashire, South West Scotland, Surrey, Warrington Halton St Helens & Knowsley, Warwickshire Solihull & Coventry, West Berkshire, West Devon, West London, West Suffolk, West Sussex, Wiltshire, Winchester, Wirral and Wycombe.

Million Women Study Co-ordinating Centre Staff:
Simon Abbott, Miranda Armstrong, Angela Balkwill, Emily Banks, Vicky Benson, Valerie Beral, Judith Black, Kirsty Bobrow, Anna Brown, Diana Bull, Benjamin Cairns, Karen Canfell, Dexter Canoy, James Chivenga, Barbara Crossley, Dave Ewart, Sarah Ewart, Lee Fletcher, Toral Gathani, Laura Gerrard, Adrian Goodill, Jane Green, Lynden Guiver, Michal Hozak, Isobel Lingard, Elizabeth Hilton, Sau Wan Kan, Carol Keene, Oksana Kirichek, Mary Kroll, Nicky Langston, Bette Liu, Maria-Jose Luque, Lynn Pank, Kirstin Pirie, Gillian Reeves, Emma Sherman, Evie Sherry-Starmer, Moya Simmonds, Helena Strange, Siân Sweetland, Alison Timadjer, Sarah Tipper, Joanna Watson, Lucy Wright, Heather Young.
